# Recent Population Dynamics of Japanese Encephalitis Virus

**DOI:** 10.3390/v15061312

**Published:** 2023-06-02

**Authors:** Jinpeng Xu, Abdul Wahaab, Sawar Khan, Mohsin Nawaz, Muhammad Naveed Anwar, Ke Liu, Jianchao Wei, Muddassar Hameed, Zhiyong Ma

**Affiliations:** 1 School of Life Sciences and Food Engineering, Hebei University of Engineering, Handan 056038, China; xujinpeng@hebeu.edu.cn; 2 Shanghai Veterinary Research Institute, Chinese Academy of Agricultural Science, Shanghai 200241, China; abdul.wahaab@uaf.edu.pk (A.W.); drsawarkhan@gmail.com (S.K.); mohsin4846@yahoo.com (M.N.); liuke@shvri.ac.cn (K.L.); jianchaowei@shvri.ac.cn (J.W.); 3 Institute of Molecular Biology and Biotechnology, The University of Lahore, Lahore 54000, Pakistan; 4 Faculty of Veterinary and Animal sciences, University of Poonch, Rawalakot 12350, Pakistan; 5 Institute of Microbiology, University of Agriculture, Faisalabad 38000, Pakistan; dr.naveed903@gmail.com; 6 Center for Zoonotic and Arthropod-borne Pathogens, Virginia Polytechnic Institute and State University, Blacksburg, VA 24060, USA

**Keywords:** Japanese encephalitis virus, population dynamic, genetic diversity

## Abstract

Japanese encephalitis virus (JEV) causes acute viral encephalitis in humans and reproductive disorders in pigs. JEV emerged during the 1870s in Japan, and since that time, JEV has been transmitted exclusively throughout Asia, according to known reporting and sequencing records. A recent JEV outbreak occurred in Australia, affecting commercial piggeries across different temperate southern Australian states, and causing confirmed infections in humans. A total of 47 human cases and 7 deaths were reported. The recent evolving situation of JEV needs to be reported due to its continuous circulation in endemic regions and spread to non-endemics areas. Here, we reconstructed the phylogeny and population dynamics of JEV using recent JEV isolates for the future perception of disease spread. Phylogenetic analysis shows the most recent common ancestor occurred about 2993 years ago (YA) (95% Highest posterior density (HPD), 2433 to 3569). Our results of the Bayesian skyline plot (BSP) demonstrates that JEV demography lacks fluctuations for the last two decades, but it shows that JEV genetic diversity has increased during the last ten years. This indicates the potential JEV replication in the reservoir host, which is helping it to maintain its genetic diversity and to continue its dispersal into non-endemic areas. The continuous spread in Asia and recent detection from Australia further support these findings. Therefore, an enhanced surveillance system is needed along with precautionary measures such as regular vaccination and mosquito control to avoid future JEV outbreaks.

## 1. Introduction

Japanese encephalitis (JE) is a vaccine-preventable disease, caused by the Japanese encephalitis virus (JEV), which is prevalent in Asian countries [1]. JEV has a positive-sense RNA genome belonging to the *flavivirus* genus within the *flaviviridae* family of five geographically and epidemiologically distinct genotypes (genotype I-V) [2]. Its genome contained 10,965 nucleotides and encoded polyprotein is further processed into three structural (capsid, membrane, envelope) and seven non-structural proteins (NS1, NS2A, NS2B, NS3, NS4A, NS4B, NS5). JEV genotype III (GIII) had been the most abundant genotype, which led to several outbreaks in JEV endemic areas until 1990. However, recent data show the emergence of genotype I (GI) as a dominant JEV genotype, and it is gradually displacing GIII. The exact mechanism of this genotype displacement needs to be explored. In nature, the virus can circulate in invertebrate and vertebrate hosts. Invertebrates (mosquitoes) act as vectors, and vertebrate hosts such as pigs and aquatic wading birds act as amplifying/reservoir hosts, and humans and equines are the dead-end hosts [3,4]. Approximately 300 million people live in Asia, where JEV is circulating endemically, and they are at risk of JEV infection. Annually, it causes 68,000 clinical cases and 10,000–15,000 associated deaths [1,5,6]. Recently, JEV cases have been controlled to a significant extent by the use of JEV vaccines all over Asian countries [7], and the demographic history of JEV has been reported in previous studies [2,8,9]. However, due to the continued spread of JEV to non-endemic areas such as Tibet, Xinjiang, Philippines, and Australia [10,11,12,13,14] and continuous detection from mosquitoes or vertebrate hosts of endemic regions [15,16,17,18,19,20,21], we felt that there is a need to reconstruct the molecular phylogeny and population dynamics of JEV using recent isolates (till December 2022) of JEV for the future perception of disease spread.

## 2. Methodology

All published and publicly available (*n* = 160) complete JEV genomes (till December 2022) were retrieved from GenBank public database (Supplementary Table S1). A MUSCLE-based multiple sequence alignment (MSA) of the data set was generated using an online tool at EBI server (https://www.ebi.ac.uk/), which was then visualized in BioEdit software [22]. The model of evolution was tested by ModelFinder tool [23], which revealed GTR + F + I + G4 as the best-fit evolutionary model for the dataset judged by Akaike and Bayesian information criterions (AIC and BIC). Timeline phylogeny reconstructions were performed in a Bayesian framework with BEAST 2 [24] using Markov chain Monte Carlo (MCMC) algorithms [25]. The GTR site model with a strict molecular clock and a fixed rate of 1.0628 × 10^−4^ mutations/site/year (calculated with BEAST2 in present study) was applied. The MCMC chain was run for 1 billion steps, with sampling of parameters every 2000 steps. Tracer v1.7.1 was used to assess the MCMC generated results, and an ESS value of >200 was considered as acceptable for all parameters of interest. The maximum clade credibility tree was extracted by TreeAnnotator, and it was then visualized and finished in FigTree (http://tree.bio.ed.ac.uk/software/figtree/).

We reconstructed a Bayesian skyline plot (BSP) [26] using BEAST 2 [24]. The BSP was generated with a strict molecular clock and a fixed rate of 1.0628 × 10^−4^ mutations/site/year. The MCMC chain was run for 1 billion steps, with sampling of parameters every 2000 steps. The ESS for all parameters of interest remained >200, as analyzed in Tracer. Finally, the BSP was visualized and extracted using Tracer v1.7.1 [27].

## 3. Results

**Figure 1:**
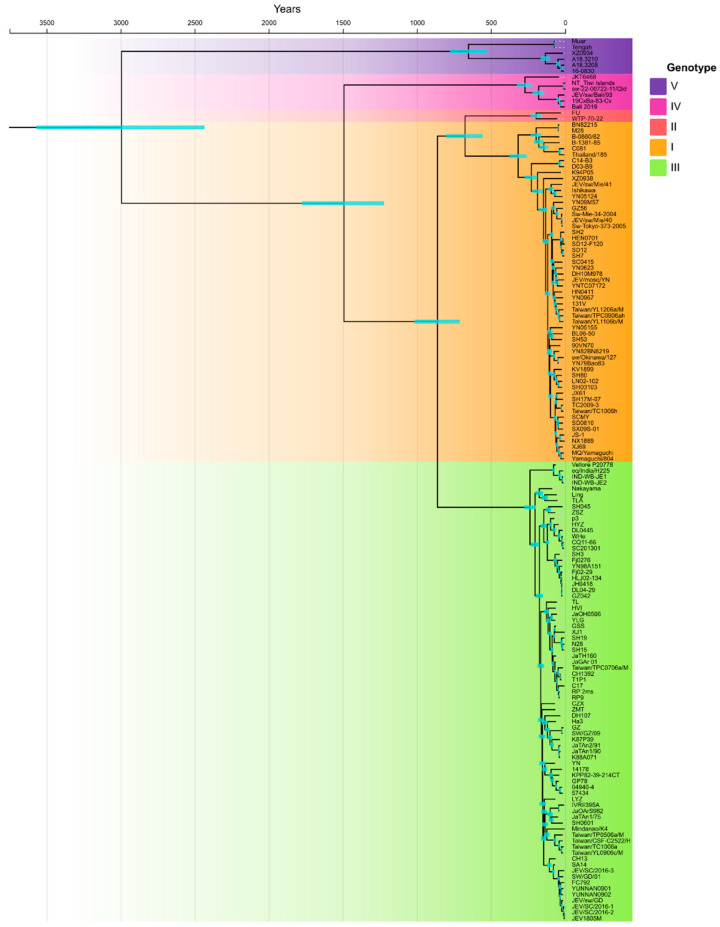
Bayesian inference-based phylogenetic tree for all available JEV complete genomes till 2022 was reconstructed in the present study. BEAST Bayesian inference-based chronograms present the divergence time estimates and phylogenetic relationship among previously reported JEV genomes. Tree was constructed based on C, E, NS2B, and NS5 protein coding sequences of all publicly available JEV sequences.

Recently, the epidemiology of JEV is changing, and it has been expanding from Asia to other regions of the world such as Papua New Guinea and Australia. The rest of the world, such as Europe, South and North America, and Pacific Islands, are also receptive due to the presence of JEV-competent mosquito vectors. To determine the continuously evolving situation of JEV, we downloaded the available complete genome sequences (*n* = 160) and constructed phylogeny and population dynamics to report the possible threat of JE disease. We constructed a Bayesian molecular timeline phylogenetic tree to investigate the most recent common ancestor (tMRCA) for all genotypes. Figure 1 explained that the tMRCA occurred about 2993 years ago (YA) (95% Highest posterior density (HPD), 2433 to 3569). The branching of the lineages occurred in the following order: genotype V, at the root of the tree; genotype IV, about 1492 YA (95% HPD, 1221 to 1776); genotype I, II, and III shared their MRCA about 861 YA (95% HPD, 709 to 1018); MRCA of genotype I and II was about 675 YA (95% HPD, 556 to 802). The mean rate of nucleotide substitution for all available (till December 2022) JEV strains isolated from a variety of hosts worldwide, estimated using a Bayesian MCMC approach, was 1.0628 × 10^−4^ nucleotide substitutions per site per year (95% HPD values 8.7795 × 10^−5^, 1.2564 × 10^−4^).

**Figure 2:**
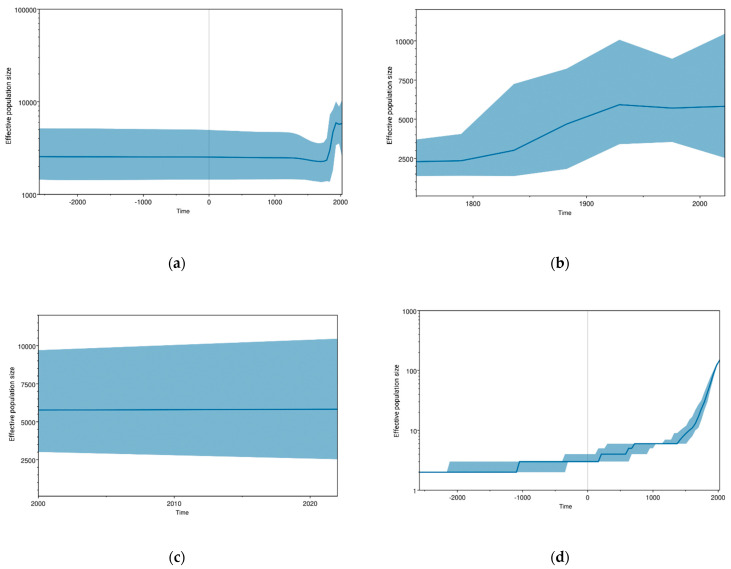
Bayesian skyline plots representing the demographic history of JEV. The central solid blue line represents the median posterior value, and the shaded area represents the 95% HPD intervals. The x-axis corresponds to time (years), while the y-axis represents the effective population size. (**a**) Population modeling during the whole evolutionary history; (**b**) Population trends during the period 1750–2022; (**c**) Population dynamics from 2000 to 2022; (**d**) JEV lineage through time.

Figure 2a–d illustrate the population dynamic of JEV. The skyline plot showed that the JEV population experienced complicated changes during the process of evolution after the 18th century. Initially, JEV remained relatively stable after its emergence (Figure 2a). However, a fluctuation was observed in the late 18th century, as shown in Figure 2b. There was a gradual rise in the JEV population observed from 1790 to 1870. It was a time when the first recurrent epidemics of JEV occurred in Japan from 1871 onwards [28]. After 1870, it continued to rise and peaked during 1920 to 1930, and a little fluctuation was seen between 1975 and 1985, as presented in Figure 2b. After that, the JEV population remained high (Figure 2b). Figure 2c explains that the JEV population is increasing slightly after 2010 to 2022, and genetic diversity has also increased slightly. The overall-JEV-lineage-through-time analysis (Figure 2d) depicted a similar pattern of JEV population dynamics to that of BSP. It showed that the JEV population remained constant in the beginning and then increased stepwise, and finally, it attained a sharp increase, whereas after the sharp increase, it makes a plateau-like pattern and maintains it to date (Figure 2b,c).

## 4. Discussion

In the present study, we performed bioinformatic analysis of the available JEV genome sequences (*n* = 160) to construct a timeline phylogenetic tree to determine the tMRCA and also determined the population dynamics of JEV. Our analysis showed that tMRCA occurred about 2993 years ago (YA) (95% Highest posterior density (HPD), 2433 to 3569). Furthermore, population dynamics analysis showed that JEV genetic diversity has been increasing since 2010. Therefore, we need to monitor JEV spread and update vaccines accordingly.

Recently, JEV cases have decreased due to the development and wide-scale application of JEV vaccines [29,30]. Although the JEV outbreak has been controlled, from this molecular data, we can conclude that disease threat still exists. Because a plateau-like pattern at high population diversity demonstrates the high genetic diversity of JEV, which infers that JEV is potentially replicating in the reservoir hosts, and it is a potential threat for future outbreaks (Figure 2c). Our skyline data show that a fluctuation was observed after 1790, and a gradual rise in the JEV population was observed from 1790 to 1870 (Figure 2b). It was a time when the first recurrent epidemics of JEV occurred in Japan from 1871 onwards [28], which is a good interpretation of our data. However, this data is not in line with previous reports where they observed the first rise in JEV population after 1930 [8]. This difference might come due to the selection of different models for analysis. However, Figure 2c illustrates that after 2000, a similar trend was observed in previous and present JEV evolutionary studies [8,9,31]. In addition, despite the continuous application of different JEV vaccines, a high level of genetic diversity infers that the virus is potentially replicating, and there are chances of gaining mutations that can lead to the emergence of new and highly pathogenic strains.

Flaviviruses such as West Nile virus (WNV), yellow fever virus (YFV), dengue virus (DENV), Zika virus (ZIKV), and JEV are the most abundant mosquito-borne viruses, causing outbreaks in different regions [32,33]. The evolutionary potential of viruses is determined by the nucleotide substitution rate [34]. In the present study, the mean rate of nucleotide substitution for JEV strains was 1.0628 × 10^−4^ nucleotide substitutions per site per year (95% HPD values 8.7795E × 10^−5^, 1.2564 × 10^−4^), which is comparable with WNV 5.06 × 10^−4^, TBEV 2.104 × 10^−4^, and YFV 4.2 × 10^−4^ substitutions per site per year [35,36,37] and shows the JEV evolutionary potential among flaviviruses. Recently, JEV GIV caused an outbreak in Australia, which highlights its potential to spread to different regions of the world and cause epidemics. JEV GIV was also detected from Indonesia [38,39]. There are multiple factors which might play a role in the spread of JEV to non-endemic areas, such as: infected mosquitoes being wind-blown transferred or harboring on planes; local mosquitoes getting JEV from infected migratory birds; and adaptive changes occurring in the virus to produce long-time viremia in vertebrate hosts, etc. Previous studies show the role of birds in the JEV zoonotic transmission as a natural reservoir and amplifying host [4,40]. Migratory birds can move hundreds to thousands of kilometers and can extend across political boundaries [41]. Bird populations are increasing because of developments in agriculture. A recent study from Korea reported that the distribution and density of migratory birds are correlated with JE cases in cities, and they might be highly potential hosts contributing to the transmittance of JEV in metropolitan areas [42]. The migration of JEV GIV-infected birds from endemic areas to non-endemic regions might have led to the spread of JEV to Australia, which requires intensive investigation.

Climatic factors can change the proliferation environment of mosquitoes, which can lead to temporal and spatial changes in mosquito density. Meteorological factors such as temperature and humidity can affect the whole life cycle of mosquitoes, accelerate or delay the growth and development of mosquito larvae, and further affect the abundance of adult mosquitoes [43,44]. The increase in the density of the mosquito population means an increase in the probability of biting the host carrying the Japanese encephalitis virus and transmitting the virus to humans [45]. Some studies found an association between JE transmission and climate changes [46]. It is important to study the impact of weather on the transmission of Japanese encephalitis, as global climate change may directly or indirectly influence the mosquito density and the virus, as well as people’s behaviors. Recently, Tu et al. reported that relative humidity has a positive impact on the transmission of JE [47]. Relative humidity influences longevity, mating, dispersal, feeding behavior, and oviposition of mosquitoes. At higher humidity, mosquitoes generally survive for longer and disperse further. Therefore, they have a greater chance of feeding on an infected animal and surviving to transmit a virus to humans or other reservoir hosts. In addition, the increase in urbanization disturbed local mosquitos’ niche and enhanced human–mosquito interaction, which might have led to an increase in the virus spread from mosquitoes to humans. There are several types of JEV vaccines such as purified, formalin-inactivated mouse-brain derived, cell-culture-derived inactivated, and cell-culture-derived live attenuated have been in use since the 1930s in endemic countries (reviewed in [48]). Many countries that experience a high JE burden, such as China, Taiwan, Japan, Taiwan, and South Korea, have reduced JE disease prevalence mainly through high vaccination coverage. Furthermore, the implementation of JEV vaccines in pigs have interrupted the JEV transmission cycle, which further contributed to the reduction in JEV burden. Overall, this shows that JE disease can be reduced significantly via large-scale vaccination in humans and intermediate/reservoir hosts.

Overall, our analysis shows that JEV genetic diversity has increased during the last few decades, which indicates the potential JEV replication in the reservoir host, which is helping it to maintain its genetic diversity and to continue its dispersal into non-endemic areas. Therefore, an enhanced surveillance system is needed along with precautionary measures such as regular vaccination and mosquito control to avoid future JEV outbreaks.

## Data Availability

No new data were created in this study.

## Supplementary Materials

The following supporting information can be downloaded at: https://www.mdpi.com/article/10.3390/v15061312/s1, Table S1: JEV isolates analyzed in this study.
