# Supplementary material for: Recent Population Dynamics of Japanese Encephalitis Virus
Source: Viruses. 2023 Jun 2;15(6):1312. doi: 10.3390/v15061312 (PMC10304036; doi:10.3390/v15061312)
Supplement: Supplementary file 1 [file viruses-15-01312-s001.zip › viruses-2402600-supplementary.pdf]

Table S1 JEV isolates analyzed in this study.

Strain	Date	Country/Region	Host	Genotype	GenBank accession no.
JaTAn1/90	1990	Japan	pig	III	AB551991
B-0860/82	1982	Thailand	pig	I	GQ902058
B-1381-85	1985	Thailand	pig	I	GQ902061
90VN70	1990	Vietnam	Human	I	HM228921
Ishikawa	1994	Japan	Mosquitoes	I	AB051292
YN82BN8219	1982	China-Yunan	Mosquitoes	I	JN381834
K94P05	1994	Korea	Mosquitoes		AF045551
M28	1977	China	Mosquitoes	I	KT957422
BN82215	1982	China	Mosquitoes	I	KT957423
KV1899	1999	Korea	Pig	I	AY316157
YN79Bao83	1979	China-Yunan	Mosquitoes	I	JN381851
Sw-Tokyo-373-2005	2005	Japan	Pig	I	AB698907
Sw-Mie-34-2004	2004	Japan	Pig	I	AB698909
131V	2007	China	Human	I	GU205163
JX61	2008	China	Pig	I	GU556217
HEN0701	2007	China	Pig	I	FJ495189
GZ56	2008	China-GuiZhou	Human	I	HM366552
HN0411	2004	China-Henan	Mosquitoes	I	JN381831.1
JEV/sw/Mie/40/2004	2004	Japan	Pig	I	AB241118.1
JEV/sw/Mie/41/2002	2002	Japan	Pig	I	AB241119.1
TC2009-3	2009	Taiwan	Mosquitoes	I	JF499788.1
YN0967	2009	China-Yunan	Mosquitoes	I	JF706268.1
BL06-50	2006	China-Guangxi	Mosquitoes	I	JF706270.1
LN02-102	2002	China-Lioning	Mosquitoes	I	JF706278.1

YN05124	2005	China-Yunan	Mosquitoes	I	JF706281.1
SD0810	2008	China-Shandong	Mosquitoes	I	JF706286.1
YN0623	2006	China-Yunan	Mosquitoes	I	JN381836.1
SH03103	2003	China-Shanghai	Mosquitoes	I	JN381847.1
SH53	2001	China-Shanghai	Mosquitoes	I	JN381850.1
JEV/Taiwan/TPC0906ah/M/2009	2009	Taiwan	Mosquitoes	I	KF667318.1
JEV/Taiwan/TC1006h/M/2010	2010	Taiwan	Mosquitoes	I	KF667321.1
DH10M978	2010	China	Mosquitoes	I	KT229573.1
YN09M57	2009	China	Mosquitoes	I	KT229574.1
YNTC07172	2007	China	Mosquitoes	I	KT957419.1
LN02-102	2002	China-Lioning	Mosquitoes	I	JF706278.1
SC0415	2004	China-Sichuan	Mosquitoes	I	JN381838.1
SH17M-07	2007	China		I	EU429297.1
SH80	2001	China-Shanghai	Mosquitoes	I	JN381848.1
SX09S-01	2009	China	Pig	I	HQ893545.1
XJ69	2007	China	Mosquitoes	I	EU880214.1
XZ0938	2009	China-Xizhang	Mosquitoes	I	HQ652538.1
YN05155	2005	China-Yunan	Mosquitoes	I	JN381852.1
JEV/sw/Okinawa/127/2012	2012	Japan	Pig	I	AB920399
JEV/MQ/Yamaguchi/2013-2	2013	Japan	Mosquitoes	I	AB981184.1
JEV/Taiwan/YL1206a/M/2012(2)	2012	Taiwan	Mosquitoes	I	KF667323.1
JEV/Taiwan/YL1106b/M/2011(2)	2011	Taiwan	Mosquitoes	I	KF667327.1
SCMY	2014	China	Pig	I	KU351668.1
JS-1	2015	China	Mosquitoes	I	KX357114.1
SH7	2016	China-Shanghai	Mosquitoes	I	MH753129.1
SH2	2016	China-Shanghai	Mosquitoes	I	MH753133.1
FU	1995	Australia	Human	II	AF217620

JKT6468	1981	Indonesia	Mosquitoes	IV	AY184212
Tengah	1952	Singapore	Human	V	KM677246
Muar	1952	Malaysia	Human	V	HM596272
WTP-70-22	1970	Malaysia	Mosquitoes	II	HQ223286
XZ0934	2009	China-Tibet	Mosquitoes	V	JF915894
JaGAr 01	1959	Japan	Mosquitoes	III	AF069076
HVI	1959	Taiwan	Mosquitoes	III	AF098735
CH13	1957	China-Sichuan	Human	III	JN381870
Nakayama	1935	Japan	Human	III	EF571853
YLG	1955	China-Fujian	Human	III	JF706280
ZMT	1955	China-Fujian	Human	III	JF706283
ZSZ	1955	China-Fujian	Human	III	JN381862
CZX	1954	China-Fujian	Human	III	JN381865
LYZ	1957	China-Fujian	Human	III	JN381869
YN	1954	China-Yunan	Human	III	JN381871
p3	1949	China	Human	III	JEU47032
Vellore P20778	1958	India	Human	III	AF080251
JaTH160	1960	Japan	Human	III	AB269326.1
JaTAn1/75	1975	Japan	Pig	III	AB551990
TL	1965	Taiwan	Mosquitoes	III	AF098737
JaOH0566/Japan/1966/human	1966	Japan	Human	III	AY508813
GP78	1978	India	Human	III	AF075723
GSS	1960	China-Beijing	Human	III	JF706275
HYZ	1979	China-Yunan	Human	III	JN381853
TLA	1971	China-Lioning	Human	III	JN381868
Ha3	1960	China-Heilongjiang	Human	III	JN381872
Ling	1965	Taiwan	Human	III	L78128
RP 2ms	1985	Taiwan	Mosquitoes	III	AF014160
RP9	1985	Taiwan	Mosquitoes	III	AF014161
K87P39	1987	South Korea	Mosquitoes	III	AY585242
CH1392	1990	Taiwan	Mosquitoes	III	AF254452

KPP82-39-214CT	1982	Thailand	Mosquitoes	III	GQ902063
JaOArS982	1982	Japan	Mosquitoes	III	M18370
SH3	1987	China-Shanghai	Human	III	JN381864
DH107	1989	China-Yunan	Mosquitoes	III	JN381873
K88A071	1988	South Korea	Mosquitoes	III	KR908703
JaTAn2/91	1991	Japan	Pig	III	AB551992
T1P1	1997	Taiwan	Mosquitoes	III	AF254453
04940-4	2002	India	Mosquitoes	III	EF623989
014178	2001	India	Human	III	EF623987
057434	2005	India	Human	III	EF623988
DL0445	2004	China-Yunan	Mosquitoes	III	JN381854
HLJ02-134	2002	China-Heilongjiang	Culicoides	III	JF706276
DL04-29	2004	China-Yunan	Mosquitoes	III	JF706272
Fj02-29	2002	China-Fujian	Human	III	JF706273
JH0418	2004	China-Yunan	Mosquitoes	III	JN381855
GZ042	2004	China-GuiZhou	Mosquitoes	III	JN381857
YN98A151	2003	China-Yunan	Mosquitoes	III	JN381861
SH045	2004	China-Shanghai	Mosquitoes	III	JN381866
Fj0276	2002	China-Fujian	Human	III	JN381867
JEV/SW/GZ/09/2004	2004	China	Pig	III	KF297916
JEV/Taiwan/TP0506a/M/2005	2005	Taiwan	Mosquitoes	III	KF667310
YUNNAN0901	2009	China	Mosquitoes	III	JQ086762
YUNNAN0902	2009	China	Pig	III	JQ086763
IND-WB-JE1	2008	India	Human	III	JX050179
IND-WB-JE2	2010	India	Human	III	JX072965
JEV/eq/India/H225/2009	2009	India	Horse	III	JX131374
CQ11-66	2010	China	Human	III	KC183732
GZ	2010	China	Pig	III	KC915016
JEV/SW/GD/01/2009	2009	China	Pig	III	KF297915
JEV/Taiwan/CSF-	2006	Taiwan	Human	III	KF667311

C2522/H/2006					
JEV/Taiwan/TPC0706a/M/2007	2007	Taiwan	Mosquitoes	III	KF667312
JEV/Taiwan/TC1006a/M/2010	2010	Taiwan	Mosquitoes	III	KF667314
JEV/Taiwan/YL0906c/M/2009(2)	2009	Taiwan	Mosquitoes	III	KF667315
JEV/sw/GD/2008	2008	China	Pig	III	KX965684
SH0601	2006	China	Pig	III	EF543861
WHe	2006	China	Pig	III	EF107523
JEV/SW/IVRI/395A/2014	2014	India	Pig	III	KP164498
SC201301	2013	China	Pig	III	KU363309
JEV/SC/2016-2	2016	China	Pig	III	KX779520
JEV/SC/2016-1	2016	China	Pig	III	KX779521
JEV/SC/2016-3	2016	China	Pig	III	KX779522
N28	2015	China-Shanghai	Pig	III	MH753126
SH1	2015	China-Shanghai	Pig	III	MH753128
SH15	2016	China-Shanghai	Mosquitoes	III	MH753130
SH19	2016	China-Shanghai	Mosquitoes	III	MH753131
SD12	2015	China-Shanghai	Pig	I	MH753127
SA14	1954	China	Mosquitoes	III	U14163
C17	2016	Angola	Human	III	KX945367.1
FC792	2017	China: Guangxi	Pig	III	MF002373.1
JEV1805M	2018	China	Human	III	MN639770.1
JEV/sw/Mindanao/K4/2018	2018	Philippines: Mindanao	Pig	III	LC461960.1
VN 113	1979	Viet Nam	Human	IV	KU705228.1
JEV/sw/Bali/93/2017	2017	Indonesia: Bali, Denpasar	Pig	IV	LC461961.1
Bali 2019	2019	Australia	Human	IV	MT253731.1
19CxBa-83-Cv	2019	Indonesia: Bali	Mosquitoes	IV	LC579814.1
JEV/Human/NT_Tiwi Islands/2021	2021	Australia: Northern	Human	IV	OM867669.1

		Territory, Tiwi Islands			
JEV/sw-22-00722-11/Qld/2022	2022	Australia: Queensland	Pig	IV	ON624132.1
A18.3210	2018	South Korea: Camp Humphreys	Mosquitoes	V	MT568538.1
A18.3208	2018	South Korea: Camp Humphreys	Mosquitoes	V	MT568539.1
16-0830	2016	South Korea: Yongsan	Mosquitoes	V	MT568540.1
C14-B3	2015	Cambodia	Pig	I	KY927817.1
D03-B9	2015	Cambodia	Pig	I	KY927818.1
JEV/MQ/Yamaguchi/804/2016	2016	Japan: Yamaguchi, Yoshida	Mosquitoes	I	LC461957.1
C081	2015	Cambodia	Human	I	KY927816.1
JEV/mosq/YN/2016	2016	China	Mosquitoes	I	MH385014.1
JEV/sw/Thailand/185/2017	2017	Thailand	Pig	I	LC461958.1
NX1889	2018	China: Ningxia	Human	I	MT134112.1
SD12-F120	2019	China	Pig	I	MN544779.1
